# Exploring the therapeutic potential of sodium deoxycholate tailored deformable-emulsomes of etodolac for effective management of arthritis

**DOI:** 10.1038/s41598-023-46119-7

**Published:** 2023-12-07

**Authors:** Gajanand Sharma, Akanksha Mahajan, Kanika Thakur, Gurjeet Kaur, Vijay G. Goni, Muniramiah Vinod Kumar, Ravi Pratap Barnwal, Gurpal Singh, Bhupinder Singh, O. P. Katare

**Affiliations:** 1 University Institute of Pharmaceutical Sciences, UGC-Centre of Advanced Studies, Panjab University, Chandigarh, 160014 India; 2 Research Scientist II, Certara UK Ltd, Simcyp Division, Level 2-Acero, 1 Concourse Way, Sheffield, S1 2BJ UK; 3 Department of Renal Transplant Surgery, Postgraduate Institute of Medical Education and Research, Chandigarh, 160012 India; 4 Department of Orthopaedics, Postgraduate Institute of Medical Education and Research, Chandigarh, 160012 India; 5 Department of Orthopaedics, East Point College of Medical Sciences and Research Centre, Bangalore, Karnataka 560049 India; 6 Department of Biophysics, Panjab University, Chandigarh, 160014 India; 7 Chitkara College of Pharmacy, Chitkara University, Rajpura, Punjab 140401 India

**Keywords:** Drug delivery, Pharmaceutics, Osteoarthritis

## Abstract

The current piece of research intends to evaluate the potential of combining etodolac with deformable-emulsomes, a flexible vesicular system, as a promising strategy for the topical therapy of arthritis. The developed carrier system featured nanometric dimensions (102 nm), an improved zeta potential (− 5.05 mV), sustained drug release (31.33%), and enhanced drug deposition (33.13%) of DE-gel vis-à-vis conventional system (10.34% and 14.71%). The amount of permeation of the developed nano formulation across skin layers was demonstrated through CLSM and dermatokinetics studies. The safety profile of deformable-emulsomes has been investigated through in vitro HaCaT cell culture studies and skin compliance studies. The efficacy of the DE-gel formulation was sevenfold higher in case of Xylene induced ear edema model and 2.2-folds in CFA induced arthritis model than that of group treated with conventional gel (p < 0.01). The main technological rationale lies in the use of phospholipid and sodium deoxycholate-based nanoscale flexible lipoidal vesicles, which effectively encapsulate drug molecules within their interiors. This encapsulation enhances the molecular interactions and facilitates the transportation of the drug molecule effectively to the target-site. Hence, these findings offer robust scientific evidence to support additional investigation into the potential utility of flexible vesicular systems as a promising drug delivery alternative for molecules of this nature.

## Introduction

Non-steroidal anti-inflammatory drugs, commonly abbreviated as NSAIDs, are the maximally accepted category of drugs for the management and alleviation of pain-associated arthritic ailments^1^. The pain-relieving and anti-inflammatory effect produced by NSAIDs is mainly attributed to the inhibition of the enzyme cyclooxygenase (COX) which play a major role in the conversion of arachidonic acid to prostaglandins^2^. At lower dose, NSAIDs exhibit analgesic activity whereas anti-inflammatory activity is reflected at relatively high doses^3^.

Amidst the class of NSAIDs, Etodolac (ETO), a novel drug having a pyranocarboxylic acid group was introduced in the 1980s with analgesic and anti-arthritic activity^4^. US FDA approved ETO in 1996 and is stated to be well condoned in patients with OA^5–7^. Chemically, ETO is 1,8-diethyl-1,3,4,9-tetrahydropyrano[3,4-*b*]-indole-1-acetic acid commonly prescribed for the treatment of acute pain and for the management of osteoarthritis and rheumatoid arthritis. It is official in the Indian Pharmacopeia of the year 2010^8^, in British Pharmacopeia of the year 2008^9^ besides in United State Pharmacopoeia. In the Biopharmaceutical Classification System (BCS), etodolac falls in the category of class II drugs defined by low solubility and high permeability having a single ionization group indicating pKa 4.65. ETO is usually preferred for the management of spondylitis, rheumatoid arthritis, ankylosing, osteoarthritis. Just like other NSAIDs, ETO acts by obstructing the pathway of the COX enzyme along with leukotriene and neutrophil activation. ETO, a preferential COX-2 inhibitor, is chemically designed in such a manner that it proves to outweigh other NSAIDs including diclofenac, ibuprofen, aspirin, and acetaminophen because of its recognized efficacy and safety profile^10^.

NSAIDs administered via the oral route of administration possess some serious adverse effects like gastrointestinal (GIT) ulcers, GIT haemorrhage, gastric and duodenal irritation, along with risks of myocardial infarction and strokes. Orally administered ETO is thus poorly tolerated due to its appropriate disadvantages. The restricted dissolving rate of ETO is presumably because to its limited solubility, in addition to this, the drug also experiences first-pass hepatic metabolism that contributes to its poor absorption. Also, the terminal half-life is 7 h demanding frequent dosing. To overcome the consequential difficulties, alternative approaches to drug administration such as the buccal, topical and nasal route, so forth can be exploited^3,11,12^.

Of late, topical route is commonly preferred due to its high degree of effectiveness and safety. Despite the numerous advantages over traditional oral drug administration, such as the avoidance of first-pass metabolism, ease of administration and prevention of systemic adverse effects or organ dysfunction, the inadequate delivery of drugs remains a significant obstacle.

In light of these shortcomings, it makes logical sense to investigate the potential of innovative drug delivery formulation techniques, such as lipoidal carrier systems, to improve delivery. These innovative delivery systems are expected to carry drug molecules to the therapeutically important site of action, namely the pain receptors, without interfering with the surrounding healthy tissues and further permitting deep permeation inside the hard, horny layers of skin, primarily stratum corneum^13–16^.

Besides, conventional topical formulations possess poor penetration to skin barriers, therefore previous literature reported the application of novel drug delivery systems to potentiate safety and efficacy of topical formulations of etodolac. These systems comprise blend of lipids, permeation enhancers and edge activators, which propagate the permeation, such as Liposomes^17^, Solid lipid nanoparticles (SLNs)^18^, Nano lipoidal carriers (NLCs)^19^, Cubosomes^20^, Film-forming spray^21^, Ethosomes^22^, Transferosomes^23^, Nanosponges hydrogel^24^, Niosomes^14^, Transethosomes^25^, Nanosuspension^26^ and Hydrophilic gel^13^. The inclusion of the edge activators, i.e., tween 20, tween 80, tween 60, span 60 and sodium deoxycholate (SDC) etc., have been found to improve the deformation ability of lipid-based systems after topical application. In a previous study the inclusion of sodium cholate or SDC as edge activators incorporated into liposomes resulted in increased transdermal absorption vis-à-vis Tween 80. Hence, SDC was observed to improve the fluidity of vesicles to spontaneously squeeze though channels in the SC and prevents vesicle rupture when crossing through the different skin layers, eventually increase the deformability of the system^27–29^.

The objective of this research is to investigate the topical delivery of ETO using SDC-decorated deformable-emulsomes (DE), a versatile vesicular system. The focus is on evaluating the permeation, drug deposition, and dermatokinetics profile of this system in order to enhance its therapeutic efficacy for pain and inflammatory conditions like osteoarthritis and rheumatoid arthritis. The developed DE was further evaluated for assessing several aspects including micromeritics, morphology, drug content, and stability tests. The DE containing ETO was subsequently subjected to various evaluations, including skin safety, skin depth profiling, and in vitro cell culture investigations. The efficacy assessment studies were conducted using a xylene-induced ear edema model and a CFA-induced arthritis paradigm on Wistar rats.

## Materials and method

Etodolac (ETO) and Phospholipon 90G (PL90G) were obtained as gift samples from M/s Ipca Laboratories, India and M/s Lipoid GmbH, Germany, respectively. Carbopol^®^ 980 (CP980; M/s The Lubrizol Corporation, Ohio, USA), Cholesterol (Chol; M/s Avanti Polar Lipids, Alabaster, USA), Capmul MCM C10 (Cap MCM C10; Abitec Corporation, Janesville, WI, USA), Cremophor A25 (Cr A25; BASF SE, Ludwigshafen, Germany), Sodium deoxycholate (SDC; M/s Loba Chemicals Pvt. Ltd., India), Ethanol, absolute (ETH; M/s Hong Yang Chemical Corporation, China) and Butylated Hydroxy Toluene (BHT; M/s S.D. Fine Chemicals Ltd., India) purchased from the respective concerns. Analytical grade chemicals and ultrapurified water were employed in the study.

### Fabrication of ETO loaded deformable-emulsomes

**Figure 1:**
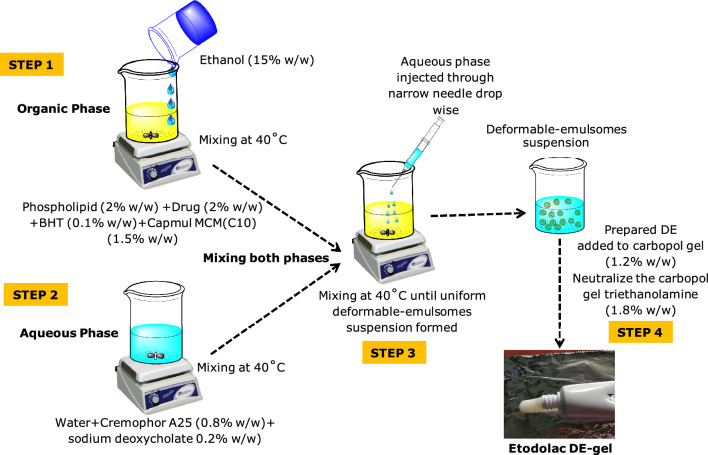
Preparation method of ETO-loaded deformable-emulsomes (DE).

The selection of the excipients was performed based on the periviuos studies of the lab (Phospholipid 90G, ETH^30^) and solubility of ETO in the excipient (Cap MCM C10 381.96 mg/mL). The other excipients were selected based on the literature review^28,29,31^. Deformable-emulsomes (DE) were formulated employing altered ethanol injection mixing method. In the first step, accurately weighed amount of drug (2% w/w; 20 mg/g of gel), PL90G (2% w/w) and Cap MCM C10 (1.5% w/w) and BHT (0.1% w/w) were dissolved in weighed amount of ETH (15% w/w) in a 100 mL beaker with continuous stirring on magnetic stirrer at 1000 rpm after complete mixing to form organic phase. In second step, edge activators viz. Cr A25 (0.8% w/w) and SDC (0.2% w/w) were added to the water medium (30% w/w) to form aqueous phase. In third step, the aqueous phase was added to organic mixture in a streamline manner under continuous high shear mixing under mechanical stirrer. Further, the prepared suspension was sonicated for 15 min, resulting in a clear homogeneous suspension of DE. The resultant concentrated suspension was preserved in refrigerated conditions in tightly-capped storage vials for future studies^32–34^. In fourth step, the DE contained 2% w/w ETO was subsequently integrated into a secondary vehicle i.e., Carbopol^®^ 980 gel 1.2% w/w (disperse 12 gm carbopol 980 in 45.4% w/w of water) and further neutralize the final gelled formulation with triethanolamine (1.8% w/w) in order to improve its suitability for topical administration^35^. The diagrammatic presentation of the method has been portrayed in Fig. 1.

### Characterization of ETO loaded DE

#### Micromeritics studies

The three most important parameters of micromeritics i.e., size of the vesicles, polydispersity index and zeta potential were measured with laser diffraction (measure hydrodynamic diameter) method, using Malvern Nanoseries manufactured by Malvern Instruments Ltd, UK. An aliquot of 1 mL of DE suspension was diluted 100 times with distilled water followed by the measurement of vesicle size and PDI. The sample was bath sonicated for 1 min using a bath sonicator at 25 °C. The zeta potential was measured of the undiluted sample at 25 °C (23.2 V/cm electric filed strength) and reported as an average value of three measurements^36^.

#### Drug entrapment efficiency and drug content

The drug entrapment was determined using centrifugation technique (Remi, RM-12C, Mumbai, India). A 2 mL microcentrifuge tube containing ETO loaded DE vesicular suspension was subjected to centrifugation at a speed of 20,000 rpm (266,866 g) for approximately 0.5 h. The clear supernatant obtained was subsequently subjected to analysis for the presence of unentrapped drug using HPLC. The quantification of the entrapped drug was achieved by subtracting the amount of unentrapped drug from the total quantity of drug that was initially added. The same procedure was carried out for emulsomes prepared without any drug (blank) to rule out interference by any other excipients. Percent drug entrapment (PDE) determined as described in Eq. (1)^37^. Similarly, the % drug content from DE-gel was determined using sonication assisted extraction with acetonitrile. Accurately weighed (proximately 0.5 gm) of DE gel was placed in a 100 mL volumetric flask. Approximately 30 mL of acetonitrile was introduced into the flask, followed by sonication and agitation for a duration of 30 min. The sonicated solution should be cooled and subsequently diluted up to the mark with acetonitrile. The sample was filtered through a 0.45 m membrane filter and analyzed using HPLC after suitable dilutions. The % drug content calculated using Eq. (2)^26^.

1 $$\text{\% Drug Entrapment}= \frac{\text{Entrapped drug in (mg)}}{\text{Total drug added (mg)}}\times 100$$

2 $$\text{\% Drug content}= \frac{\text{Measured amount drug in DE-gel}}{\text{Theoretical amount of drug in DE-gel}}\times 100$$

#### Vesicle count

A 10-time dilution of ETO loaded DE was prepared in distilled water and kept on Haemocytometer grid (Neubauer, Fein-Optik, Germany). The vesicle density per volume was determined using microscope. The number of vesicles in 144 small squares were counted and Eq. (3) was further used to calculate the number density^35^.

3 $$ {\text{ No}}{\text{. of vesicles per cubic mm }} = \frac{{{\text{No}}{\text{. of}}\,vesicles\,{\text{ in small squares x Dilution factor }} \times { 4000}}}{{{\text{Total no}}{.}\,{\text{of}}\,{\text{small squares}}\,{\text{ counted}}}}. $$

#### Deformability index

Deformability index of ETO-loaded DE was measured employing vesicle-extruder (Eastern Sci. Inc., USA). A vesicle extruder was used to pass the vesicular suspension through polycarbonate membrane filter with a size of 50 nm. The size of vesicles was measured initially and then after the extrusion process using Malvern Zeta Sizer Nanoseries. The deformability index was calculated using Eq. (4)^29,35^.

4 $$\text{Percent Deformation}= \frac{(\text{PE-PA})}{\text{PA}}X100$$

PE is particle size measured before any extrusion (n = 3) and PA is average particle size measured after the process of extrusion (n = 3).

### Morphological evaluation

The topographical and morphological traits of DE were assessed using Field Emission-Scanning Electron Microscope (FESEM; Hitachi SU8010, Japan). A drop of DE suspension was placed on a carbon coated copper grid for 15–30 min for drying which were further coated with gold. All samples were examined at an accelerated voltage of 5.0 kV and magnification of 220× and 20,000×^38,39^.

### Calorimetric studies

Differential scanning calorimetry (DSC) curves of ETO, PL90G, Cap MCM C10, Cr A25), SDC and DE formulation were noted using DSC Q20 TA (M/s PerkinElmer Inc. USA). 5 mg of the sample was weighed into a hermetically sealed pan made of aluminium and further heated at 10 °C/min over 20˚C and 280˚C temperature range. The flow of nitrogen was maintained at 22 mL/min. Each sample was measured for three runs. The DSC curves were further studied using DSC software(s) (i.e., Star^e^ and Universal) and scans were recorded^40^.

### Infra-red spectroscopy

Infrared spectra of ETO, PL90G, Cap MCM C10, ETH, Cr A25), SDC and DE formulation were determined employing (Spectrum Two™ M/s PerkinElmer Inc., USA) at temperature of 25 °C and wave number 3500 cm^−1^–500 cm^−1^. Potassium bromide (KBr) was mixed with the sample in the ratio of 2:98 to prepare a pellet^41^. FTIR measurements were carried out using potassium bromide (KBr) technique where small amount of the each samples were dispersed with KBr to form a pellet followed by spectra acquisition in transmission mode^42,43^.

### Ethics approval and consent to participate

Confirm that all the experiments were performed in accordance with Institutional Animal Ethics Committee, Panjab University, Chandigarh, INDIA (Ref. letter No. PU/45/99/CPCSEA/IAEC/2020/469 in 2020).

## Evaluation of ETO loaded-DE gel formulation

### pH measurement

pH of prepared DE-gel was investigated using pH meter (Cyber Scan, Eutech Instruments Pte Ltd., Singapore). The assessments were carried out three times and the average numbers were premeditated^26^.

### Rheological studies

The rheological study of DE-gel formulation was done using cone and plate type rheometer (Rheolab QC, Anton Paar GmbH, Vienna, Austria). The rheology measurement was carried out at a varying shear rate increased from 0 to 100 s^−1^ at temperature of 30 °C^44^. The relationship between shear stress (τ) and shear rate (γ) was analysed using Herschel-Bulkey Model as represented in Eq. (5)^45^.

5 $$ \tau = \tau_{0} + {\text{ k}}\gamma^{{\text{n}}} . $$

In the given equation, k represents the consistency index, τ_0_ denotes the yield stress, and n signifies the power law-exponent.

### Texture analysis

To determine other rheological features, i.e., consistency, firmness, cohesiveness (stickiness) and viscosity index of the formulations texture profile analysis of DE gel was performed by employing TTC spreadability rig fitted on Texture Analyzer™ (M/s Stable Micro Systems Ltd., UK)^42,46^.

### Ex vivo permeation studies on mouse skin

The permeation experiments were performed employing using a diffusion assembly prepared *in-house*. The apparatus is composed of two reservoirs including donor and receiver compartment along with the port where sampling is done. The skin tissue was clamped on the hollow tube end exposed to the receptor phase lying underneath. The skin from a single rat was used for six diffusion cell assembles to avoid variations in the data. Weighed quantity (~ 0.5 g) of DE-gel and conventional gel, containing ETO equivalent to 2% w/w were introduced uniformly into the donor compartment. The cross-sectional mean surface area available for diffusion was 3.15 cm^2^ and receptor volume was 40 mL. The receiver media with a composition of phosphate buffer saline (pH 7.4) and ETH (80:20, %v/v) was stirred at 200 rpm. ETH was incorporated in the diffusion medium to maintain the sink condition^30^. The temperature of the receptor medium was maintained at 32 ± 0.5 °C by warm water in the outer jacket (i.e., glass beaker) of the cells employing a thermostatically controlled magnetic stirrer to equilibrate the system. 1 mL volume of samples were removed at suitable intervals for a period of 24 h and replaced with fresh media to maintain the receptor volume. The collected samples were subsequently diluted and subjected to quantitative analysis utilizing HPLC^47^.

### Drug deposition studies in skin

After 24 h of skin permeation studies, the mounted tissue was carefully separated from the diffusion cell assembly. Then skin tissue was washed three times with triple-distilled water and further dried with cotton swab. Later, the tissue was then cut in to small fragments and mixed with 5 mL ACN and shaken for 12 h at 32 ± 1 °C to completely extract ETO. After 12 h supernatant was removed and filtered and subjected to drug content analysis (in triplicate) by using HPLC^48^.

### Skin depth profiling using confocal microscopy

The dye loaded formulation was prepared by method as adopted in Sect. “Fabrication of ETO loaded deformable-emulsomes” except drug is replaced with coumarin-6 at a concentration of 0.15 µmol/mL. Dye loaded DE formulation and coumarin-6 dye alone were applied for 6 h to the rat skin followed by rinsing the skin with phosphate buffer (pH 7.0) and the fixed slides were prepared. Fluorescence in different skin layers was visualized with Laser Scanning Microscopy (CLSM; Nikon Eclipse Ti M/s Nikon Instruments Inc., Melville, USA)^42,49^.

### Dermal kinetic studies

Dermatokinetic analysis of developed DE-gel formulations was carried out on excised skin tissue from Wistar rats in comparison with conventional gel. The tissue was mounted on diffusion cell assembly (in house assembly). Weighed quantity (0.5 g) of DE-gel, was introduced uniformly into the donor chamber. The receptor compartment had similar composition as previously explained in ex vivo permeation studies. The assembly was continuously stirred for 12 h so that sink conditions are maintained. The mounted skin was collected from each diffusion cell at subsequent time intervals and removed skin was washed three times to remove any formulation residue. Then removed skin was dipped in hot water (60–80 °C) for 3–5 s, to detached epidermis from dermis. The detached skin layers are further cut in to fragments and mixed with 5 mL ACN and kept for 12 h at 32 ± 1 °C to completely extract ETO. Drug content was evaluated (as discussed earlier) in both epidermis and dermis layers^42,50^.

Data obtained from above was fitted in one-compartment model according to equation below Eq. (6):

6 $${C}_{Skin} = \frac{Kp. {C}_{max}^{Skin}}{(Kp-Ke)}\left({e}^{-Kpt}- {e}^{-Ket}\right).$$

In equation, C_skin_ is the drug concentration present in skin at time t, K_p_ is the permeation constant, ${C}_{max}^{Skin}$ is the maximum drug concentration reached in skin, K_e_ is the skin drug elimination constant. These dermatokinetic parameters K_p_, ${C}_{max}^{Skin}$, ${T}_{max}^{Skin}$ (time required to achieve ${C}_{max}^{Skin}$) are computed using excel sheet software and for area under the curve (AUC_0–12 h_)^50,51^.

### In vitro cell culture analysis

The aim of conducting in vitro cell culture analysis in topical formulations is to assess the potential effects of the formulation on cultured cells, providing insights into its compatibility, safety, and potential efficacy. This analysis helps in understanding cellular responses, absorption rates, and possible cytotoxicity, aiding in the evaluation and refinement of the formulation's characteristics for effective and safe application.

#### Cell culture and growth conditions

Cell line studies were carried out using human epidermal keratinocyte cell lines (HaCaT) obtained from NCCS, Pune, India. HaCaT cell line were grown in the keratinocyte serum free (KSF) medium and further it was placed in incubator at 37 °C containing 5% CO_2_ gas in the environment. The cultured growth cells were again washed with phosphate buffer pH 7.4 solution free from any calcium/magnesium ions. The cell layers were further separated by the addition of 2.0 mL of 0.53 mM EDTA solution with 0.05% w/v of trypsin. The separated cells were diffused smoothly by pipetting into the fresh KSF medium with 10% fetal bovine serum. Further, suspension containing cells was subjected to centrifugation at 125 g and 4 °C for 5 min. The sedimented cells were further suspended into fresh KSF medium at a density of 10,000 cells per well in 96-well plates and 50,000 cells per well in 6-well culture plate and incubated in the CO_2_ incubator^42^.

#### MTT assay

The investigation focused on the examination of cell proliferation using a modified MTT (3-(4,5,-dimethylthiazole-2-yl)-2,5-diphenyltetrazolium bromide) assay. The HaCaT cells, namely the lx104 cells, were cultured in KFS medium within 96-well plates. The cell suspension was subjected to treatment with a pure drug solution (consisting of a hydro-alcoholic solution) as well as a suspension of DE (made in KSF medium). The control cells were administered exclusively with inert substances, namely a hydro-alcoholic solution and a blank DE suspension. Following the application of the solution treatment, the cells were subjected to incubation within a controlled incubator environment at a temperature of 37 °C for a duration of 48 h. The cells were undergoing washing step using a phosphate buffer. Following that, a measured portion of 150µL of a sterile MTT solution (2.5 mg/mL in phosphate buffer saline) was introduced into every well. The culture plates were subjected to stirring for a duration of 2 h at a temperature of 37 °C using a plate-shaker. Following this, the plates were placed in a CO_2_ incubator for incubation. The plates underwent centrifugation at a force of 1200 g the acceleration due to gravity for a duration of 15 min. The liquid portion was discarded, while the MTT formazan crystals were solubilized in 200µL of dimethyl sulfoxide (DMSO). Additionally, a stirring process was conducted for a duration of 20 min, during which the optical density (OD) was assessed at a wavelength of 570 nm^52,53^.

#### Determination of cellular uptake

For determination of cellular uptake of coumarin-6 dye loaded DE suspension by HaCaT cell lines, lx104 cells were seeded on a 12-well cell culture plate in keratinocyte growth medium (Gibco). The cell culture medium was substituted with mixture of media and the dye loaded DE suspension (20 µg/mL) incubated for 3 h at 37 °C. Before imaging, cells were washed 3 times with 1 mL of PBS. Hereafter, the cells were fixed with 4% paraformaldehyde solution and were viewed under florescence microscope (EVOS) under GFP and blue filter^49^.

## Stability studies

### Chemical stability

The final formulation is a gel, henceforth only the final formulation was placed for the stability studies. The stability of the developed DE-gel was performed according to ICH guidelines at controlled temperatures i.e., 25 °C ± 2 °C/60% ± 5% RH and at 40 °C ± 2 °C/75% ± 5% RH for 6 months to examine the shelf-life and storage condition of the prepared formulation^54^.

### Physical stability

The physical stability of DE-gel was examined in order to study potential changes in organoleptic characteristics after storage. The DE-gel formulations were placed at controlled room temperature of 25 °C ± 2 °C/60% ± 5% RH and at 40 °C ± 2 °C/75% ± 5% RH storage conditions for 6 months to examine the physical change (like discoloration), odour change, crystal formation, consistency of gel, phase separation and change in pH^54^.

## Efficacy assessment on animal model

### Animal ethical compliance

All the experimental related protocols were duly approved by Institutional Animal Ethics Committee, Panjab University, Chandigarh with wide Ref. letter No. PU/45/99/CPCSEA/IAEC/2020/469 in 2020.

Confirm that all the experiments were performed in accordance with Institutional Animal Ethics Committee, Panjab University, Chandigarh, India.

Confirms that the authors complied with the ARRIVE guidelines.

### Skin compliance studies

Female Balb/c mice were used to determine the patient skin-friendly properties of the DE-gel *vis-à-vis* conventional product using Modified Draize patch test. To avoid any probability of scepticism, female BALB/c mice were categorised into three groups, with 4 animals in respective groups (n = 4). Group A dermal application of normal saline which served as untreated group; Group B dermal application of conventional gel formulation and Group C dermal application of DE-gel formulation.

To initiate the procedure, firstly hairs were plucked off aiding a 0.15 mm animal hair clipper, faced on the skin dorsal side. Around 0.5 g of both the gel formulations were applied uniformly on the clipped off area of mice skin. Furthermore, the tissue was evaluated on the basis of physical examination as well as the histopathological assessment. In the former any visual changes like redness was observed for a period of 7 days after its application measuring the average erythemal grades ranging from 0 to 4 recorded in accordance with the severity of erythema viz. 0 = erythema absence, 1 = Slight observation of erythema (light pink), 2 = Moderate erythema (dark pink), 3 = Moderate to severe erythema (light red), 4 = Severe erythema (dark red)^43,55^.

### Anti-inflammatory assessment

#### Xylene-induced ear edema model

The activity was carried out on male BALB/c mice to study the effect of xylene-induced ear edema model. The procedure initiates by inducing cutaneous inflammation using xylene in conscious male BALB/c mice via topical route. Prior to the application of the irritant, the gel formulations were administered 30 min in advance during the testing process. The animals were categorized into three discrete groups (n = 4 per group). Group A: dermal application of normal saline (control); Group B: dermal application of conventional gel and Group C dermal application of DE-gel formulation^56,57^.

The irritant was injected with a micropipette (50 µL/ear) to the interior of the right ear and the left ear represented as control. After 30 min of Xylene application, the mice were immolated by cervical dislocation and the two of the ears (left and right) were expunged instantaneously, by creating parallel cuts throughout the surface of the ear in a hollow pattern. After excision, both the ears were measured and swelling percentage was computed as in Eq. (7):

7 $$ \% \,Swelling\, = \,\left( {\frac{B - A}{A}} \right) * 100, $$

where, A as weight of left ear and B as weight of right ear.

#### Anti-arthritic activity: CFA induced Arthritis in Wistar rats

To assess the anti-arthritic activity, CFA induced arthritis model in female Wistar rats is the generally adopted method. The procedure initiates by division of rats into four groups (n = 4 per group). Group A: dermal application of normal saline (untreated); Group B: served as diseased control. Group C: dermal application of conventional gel and Group D: dermal application of DE-gel formulation. Here the animals were anesthetized using diethyl ether and 100 µl of CFA was injected via into the right hind foot pad Wistar female rat on day 1 intradermally. Hereafter, the animals were observed for arthritis each day. The CFA holds 1 mg of heat killed bacterium in each mL along with 0.85 mL paraffin oil and 0.15 mL mannide mono-oleate. The therapeutic course of medication begins on the 8^th^ day after the complete introduction of arthritis and further it was sustained for two weeks. Both the novel DE-gel and conventional were applied once daily on knee joints and paws of respective group of animals^56,58^.

#### % Arthritis swelling reduction

At various time intervals, thickness of paw was measured for each animal^58,59^. Evaluation of each paw for inflammation and arthritis-swelling reduction was measured as given in Eq. (8):

8 $$\text{\% Arthritis swelling reduction=}\frac{\left(\text{Paw thickness each day-Paw thickness control}\right)}{\text{Paw thickness control}}\times 100$$

#### Histopathological studies

In order to document any modifications in the skin and paw joint of an arthritic rat model injected with CFA and treated with drug formulations, histopathological investigations were conducted. Following the sacrifice of the animals, skin samples were collected, processed accordingly, and stained with haematoxylin and eosin. The processed skin samples were then examined under a microscope to identify any histopathological alterations^55,60^.

### Statistical analysis

A two-way analysis of variance (ANOVA) is used on the data, and then Tukey's multiple 16 comparison test is performed. Statistical significance had been selected to apply when the p < 0.05, unless otherwise noted.

## Results and discussion

### Characterization of the DE formulation

#### Micromeritics

**Figure 2:**
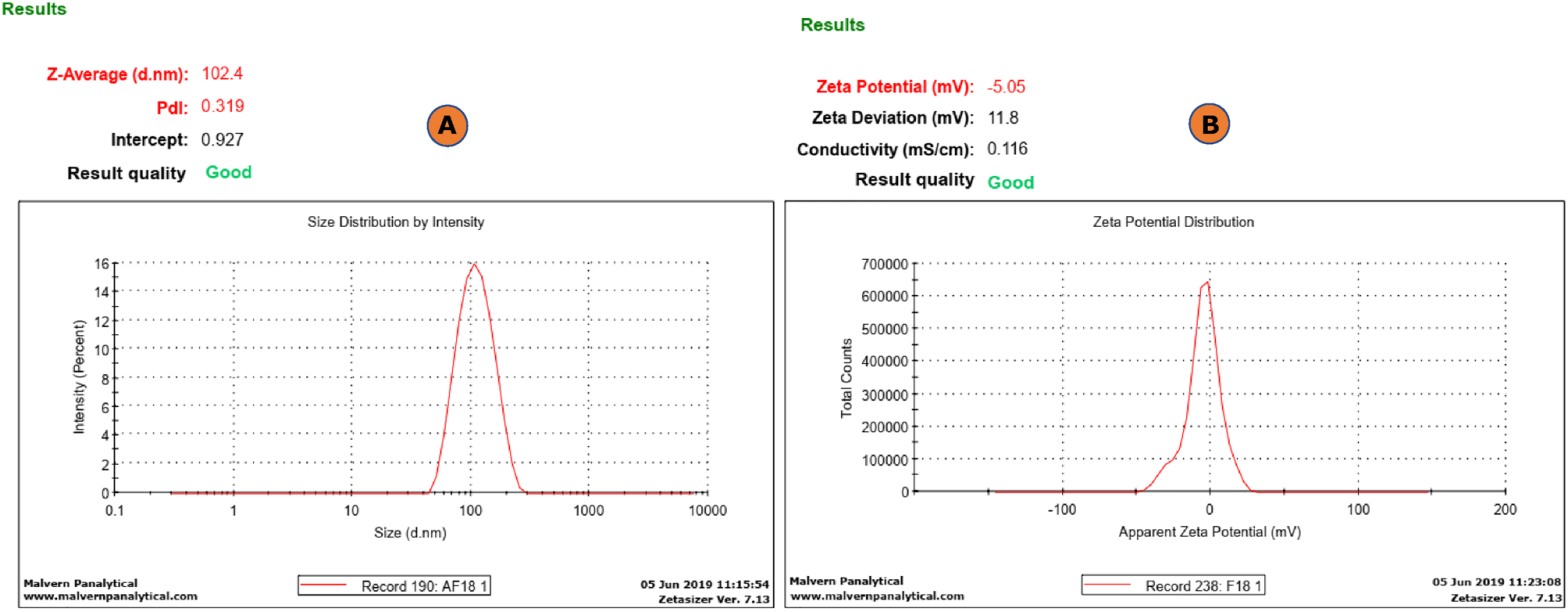
(**A**) Depicting vesicles size distribution and PDI; (**B**) Zeta potential of deformable-emulsomes.

The size of vesicles is a crucial factor that significantly influences the ability of vesicular systems to penetrate the skin. Fig.  2A illustrates the vesicle size distribution of the DE formulation. The mean vesicle size of the DE was found to be 102.4 ± 8.9 nm. The PDI of the vesicles was found to be 0.319 ± 0.016 (less than 1), promising constricted distribution of the polydispersed phase. The zeta potential is a critical factor in assessing the stability of formulations and the capacity of drug delivery systems to adhere to the biological surfaces. The zeta potential of the prepared DE formulation was determined to be 5.05 ± 2.15 mV, as illustrated in Fig. 2B. The present formulation is comprised on non-ionic surfactants and such systems are not stabilized by electrostatic repulsions as in the cases with the dispersion with zetapotential ≥  ± 25 mV. Such systems are also stable owing to steric repulsion. However, the final formulation was a gel, therefore this zetapotential was sufficient enough for the stable nano dispersion gelled in a carbomer-based hydrogel^61–63^.

#### Drug entrapment efficiency and drug content

The percentage drug entrapment from the DE vesicular suspension was found to be 81.87% and the drug content of the DE-gel was found to be 100.04%. This high drug entrapment might be plausible due to the presence of ethanol and cap MCM C10 that would have resulted in better solubilization for the drug and its localization within the interiors of the vesicles.

#### Vesicle count

To find the population of vesicles in prepared formulation is important parameter for drug entrapment. It was found that the density of un-sonicated DE vesicular population was found to be 242,500 vesicles/mL. The findings confirmed that the sufficient vesicles were formed to enclose the active drug compound.

#### Deformability index

The deformability index value of DE formulation was found to be 0.91. The optimized DE formulation was found to cross a pore around 2-times smaller to the intact sizes. This property ratified the selection of right edge activator i.e., SDC and ETH in the concentrations to obtain biologically permeable vesicles. The DE of 102.4 nm size has quite sufficient flexibility (i.e., ~ 2 times) to cross the skin barrier pores. The ideal size of the vesicular carriers to cross the skin barriers is around 80–120 nm^64^. Henceforth, the developed system can cross the skin barriers without compromising on shape/losing drug load. Due to inherent deformability of the vesicles, selection can be justified for efficient epicutaneous delivery of ETO. The drug entrapment after extrusion was also determined and the values were non-significantly different than the non-extruded system.

### Morphological evaluation

**Figure 3:**
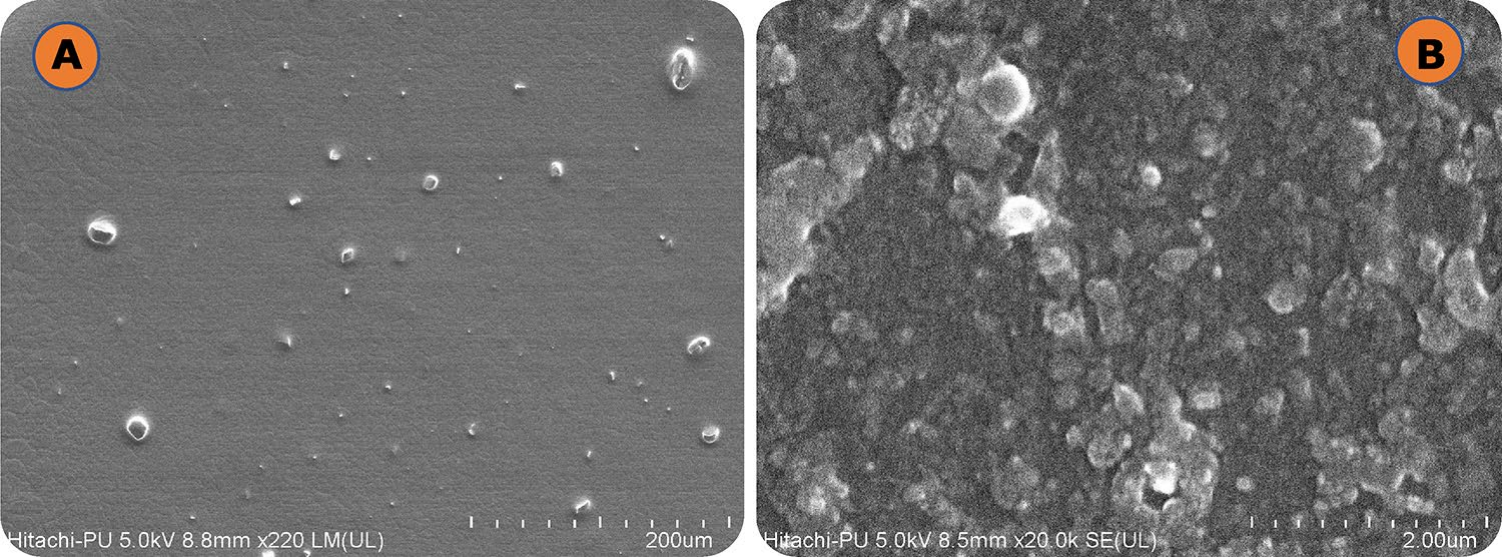
FESEM photomicrographs of DE formulation at (**A**) 220 × and (**B**) 20,000x.

The study employed field emission scanning electron microscopy (FESEM) to investigate the morphology and aggregation of DE, as well as its surface structure and morphological characteristics. The DE suspension was observed using a three-dimensional visualization technique, which revealed that the particles had a spherical shape, demonstrated polydispersity, and did not exhibit any evidence of aggregation, as shown in Fig. 3A (at magnification of 220 ×) and Fig. 3B (at magnification of 20,000 ×).

### Calorimetric studies

**Figure 4:**
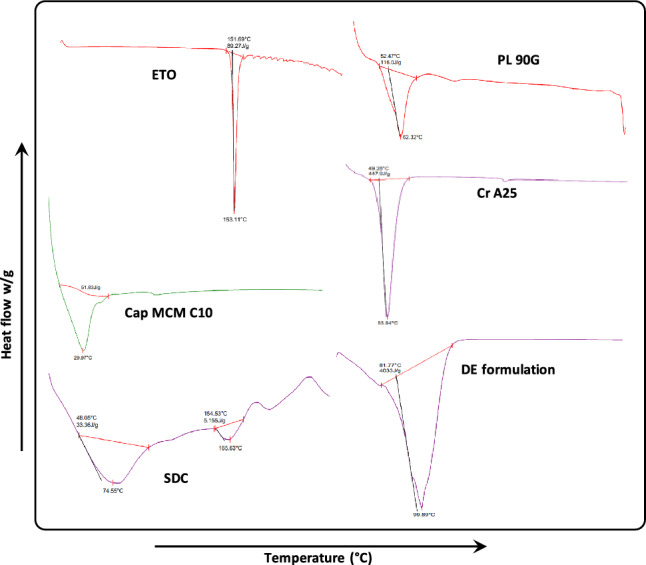
DSC thermogram of etodolac (ETO), Phospholipon 90G (PL 90G), Capmul MCM C10 (Cap MCM C10), Cremophor A25 (Cr A25), Sodium deoxycholate (SDC) and Deformable-emulsomes (DE).

The fusion characteristics of crystalline materials were examined using the differential scanning calorimetry (DSC). The drug (ETO) displayed a sharpened endothermic peak at a temperature of 153.11 °C, which closely corresponds to the published melting point of the drug (i.e., 145-143 °C). The observed melting behaviour indicates that the drug is in a crystalline state rather than an amorphous state. The other excipients like PL 90G, Cap MCM C10, Cr A25 and SDC exhibited respective endothermic peaks at 62.32 °C, 29.97 °C, 55.84 °C and 74.55 °C corresponding to their reported melting point^40,65–67^. Likewise, the developed DE formulation displayed an endothermic peak at 99.89 °C and the disappearance of drug peak may be ascribed to the encapsulation of drug in the interiors of the developed system. The respective DSC thermograms of are depicted in Fig. 4

### Infra-red spectroscopy (FT-IR)

**Figure 5:**
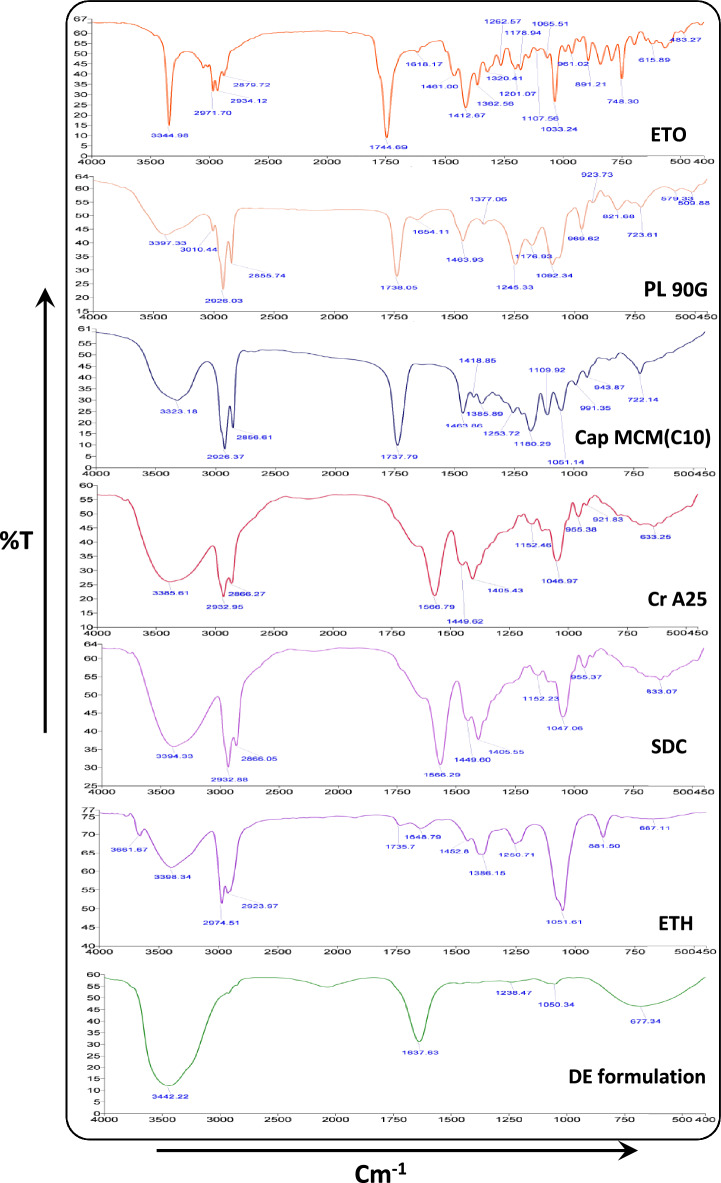
FT-IR spectra of etodolac (ETO), Phospholipon 90G (PL90G), Capmul MCM C10 (Cap MCM C10), Cremophor A25 (Cr A25), Sodium deoxycholate (SDC), Ethanol (ETH) and Deformable-emulsomes (DE).

The drug (ETO) and compatible excipients like PL 90G, Cap MCM C10, Cr A25, SDC, ETH and DE formulation spectra have been depicted in Fig. 5. The major peaks of ETO alone were recorded at 3344 cm^−1^, 1744 cm^−1^, 1618 cm^−1^, 1461 cm^−1^, 1262 cm^−1^ and 1033 cm^−1^ which confirms the identity of drug specimen as per recognized standards. The primary peaks of PL 90G were detected at 3397 cm^−1^, 2926 cm^−1^ and 2855 cm^−1^, 1738 cm^−1^, 1654 cm^−1^, 1463 cm^−1^. Cap MCM C10 peaks were observed at 3323 cm^−1^, 2926 cm^−1^, 1737 cm^−1^, 1463 cm^−1^, 1180 cm^−1^. The peaks of Cr A25 were reported at 3385 cm^−1^, 2932 cm^−1^, 1566 cm^−1^, 1449 cm^−1^. SDC peaks were measured at 3394 cm^−1^, 2932 cm^−1^, 1566 cm^−1^, 1449 cm^−1^, 1047 cm^−1^. The ETH spectra were recorded at 3398, 2974, 2923, 1386. In the IR spectra of the DE, the major peaks of ETO were masked because of overlapping of the drug peaks with excipient ones. The formulation showed majority characteristic peaks with no shift and reduced intensity. This corroborates there is no interaction between drug and excipients.

## Evaluation of ETO loaded-DE gel formulation

### pH measurement

The pH of developed DE gel was found to be 6.2, which is close to skin i.e., 4.5–6.4 as per literature. As pH of the formulation is neutral or closer to the pH of the skin, the formulation can be considered as safe or may not cause any skin irritation on application.

### Rheological studies

**Figure 6:**
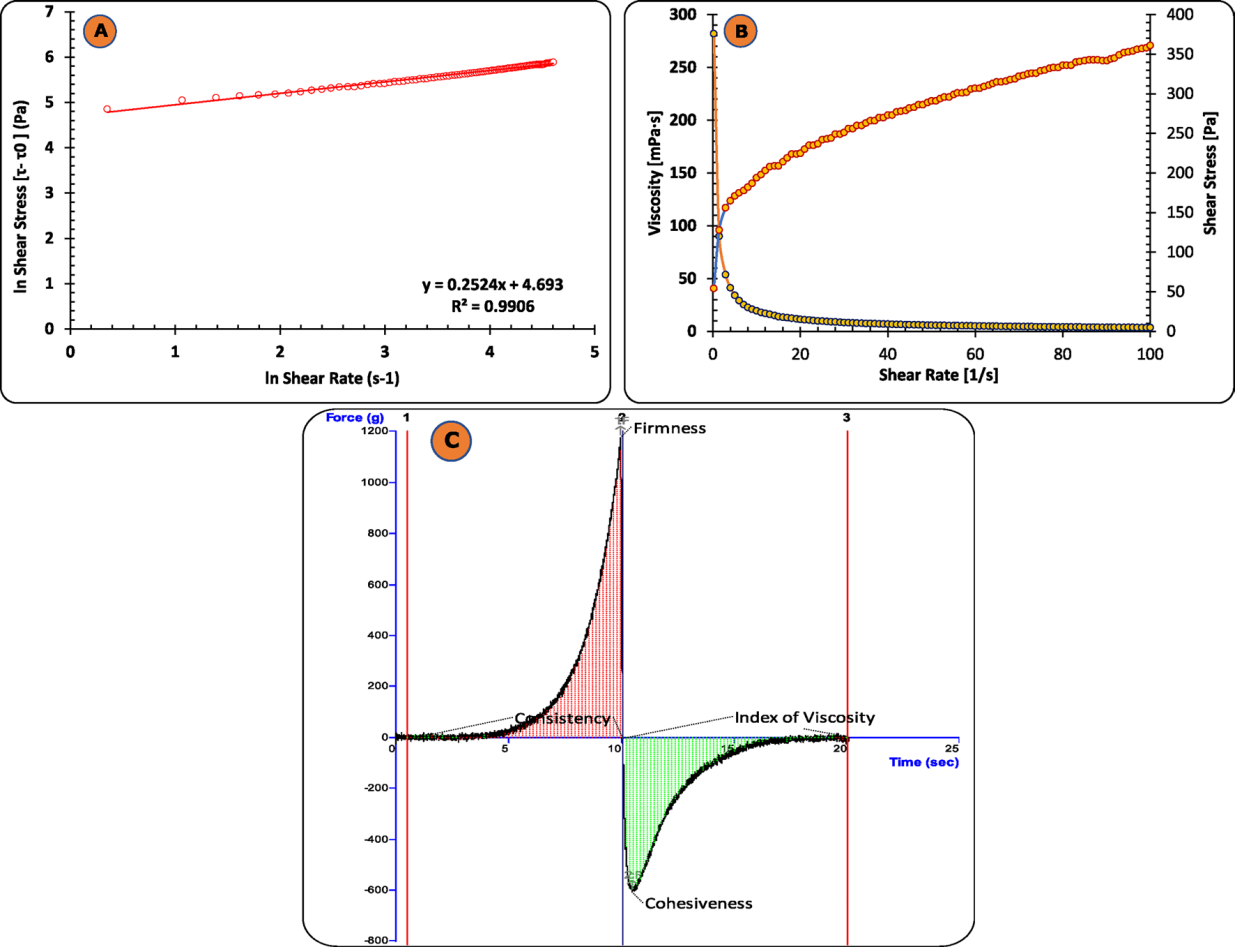
(**A**) Graph of the Herschel-Bulkey model showing the relationship between the natural logarithm of shear stress and the natural logarithm of shear rate; (**B**) Plot of shear rate vs viscosity and shear stress for the DE-gel formulation; (**C**) The texture analysis of the developed DE-gel formulation.

The micro-mechanical properties of gels can be assessed by viscometric analysis. The flow properties of DE-gel were recorded by using cone and plate viscometer and the values of parameters were computed using Herschel-Bulkley model. The value of flow index (n = 0.252), obtained from the slope (Fig. 6A), is less than 1 which indicates DE-gel formulation observed a pseudoplastic (shear-thinning) behavior, thus, reflecting decreased formulation viscosity with increase in shear rate (Fig. 6B). The appearance of pseudoplastic behaviour can be attributed to the underlying colloidal network structure, which exhibits deformation and adjustment in response to the direction of flow. The flow behaviour observed in this context might be associated with the existence of microstructures within a three-dimensional lattice network. The consistency index K (Pa.s^n^) and yield value were found to be 109.18 Pa and 120.55 respectively, which could be due to gel structure rigidity within the hydrogel which required more force to initiate its flow. However, the intrinsic viscosity of DE-gel was found to be 106.7 Pa.s. Attaining a specific phase and viscosity is a crucial prerequisite in the formulation of DE-gel to facilitate their convenient transportation and storage at an optimal temperature.

### Texture analysis

Figure 6C and Table 1 both represent the textural curve along with the various values of textural parameters such as firmness, consistency, cohesiveness, and index of viscosity that were acquired from texture analysis of the developed DE-gel formulation. The visual illustration of the DE-gel formulation shows better gel strength, suggesting greater capability to hold the gel at the topical site for a longer period of time, exhibiting smooth extrudability, and ensuring that the gel is easy to spread.

**Table 1:** Shows the data for the various parameters obtained from the texture analysis of the developed DE-gel formulation.

Formulation	Firmness (g)	Consistency (g.sec)	Cohesiveness (g)	Index of viscosity (g.sec)
DE-gel formulation	1176.25	1680.46	− 608.97	− 1418.33

### Ex vivo permeation and drug deposition studies on mouse skin

**Figure 7:**
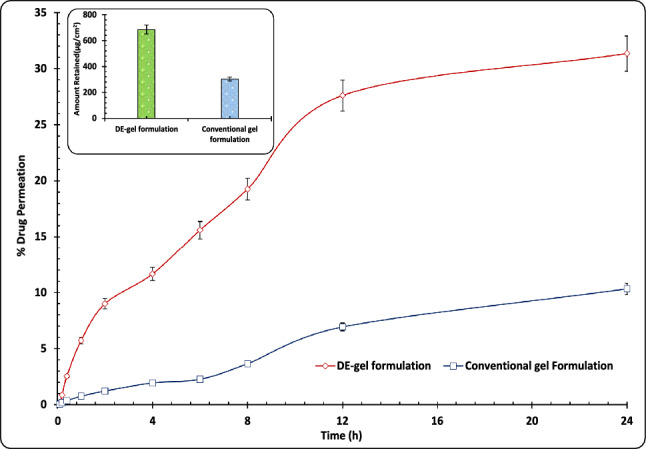
Ex vivo permeation profile of DE-gel and conventional gel formulations. Each bar indicates ± SD (n = 3). Inset figure depicting bar diagram for the amount of drug deposited by the skin for DE-gel and conventional formulations. Each cross bar indicates ± SD. (n = 3).

The final DE-gel was finally studied for desired attributes with regard to permeation of ETO across skin *vis-à-vis* the conventional gel of equivalent strength. The data presented in Fig. 7 demonstrates the permeation profiles of two formulations. It is evident that the developed DE-gel formulation exhibiting 31.33% drug permeation across of the rodent skin, exhibited superior transport properties compared to the conventional gel formulation (10.34% ETO permeation). This study provides evidence for the better transport properties of the newly developed carrier-based formulation in comparison to the conventional formulation. The enhanced penetration of ETO from the DE-gel formulation can be attributed to the phospholipid's compatibility with the skin, which is in turn attributable to the effective drug movement properties of the vesicular carriers. Similar findings with phospholipids have been frequently reported and these make the phospholipids are one of the important constituents of the topical formulations^68^. Figure 7 Inset depicts the results of drug deposition in skin reveal that the DE-gel formulation exhibits 648.68 µg/cm^2^ (i.e., 33.13%) and conventional gel exhibits 304.08 µg/cm^2^ (i.e., 14.71%). DE-gel formulation is 2.25 times significantly higher drug deposition than that of conventional gel (*p* < 0.01). This suggested DE-gel effectively makes the drug more readily available within the different dermal layers, also depositing them within target sites by means of closely integration of phospholipids to the lipids of the skin.

### Skin depth profiling using confocal microscopy

**Figure 8:**
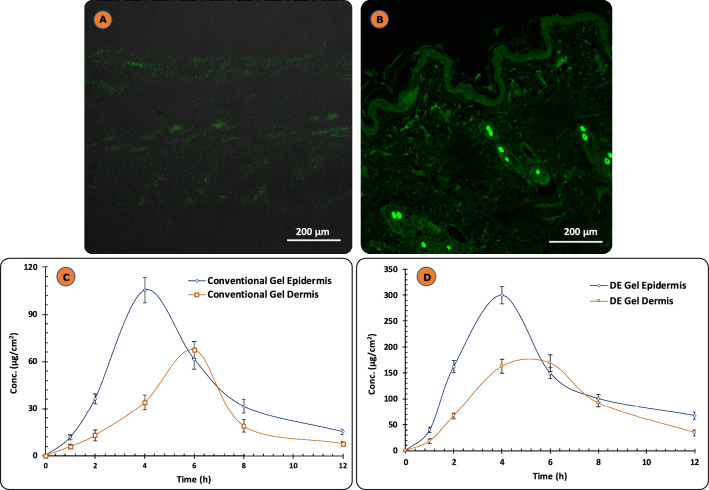
(**A**) CLSM of coumarin-6 dye alone; (**B**) coumarin-6 dye loaded-DE formulation in the deeper layer of the skin; (**C**) ETO concentration time profile in epidermis and dermis of Wistar rats after single application of Conventional gel formulation; (**D**) ETO concentration time profile in epidermis and dermis of Wistar rats after single application of DE-gel formulation. Each cross bar indicates ± SD (n = 4).

The CLSM technique was used to determine the extent and pattern of DE formulation penetration into the skin layers. The efficacy of a coumarin-6 dye loaded-DE formulation was assessed via topical administration on rat skin for a duration of 6 h. The Fig. 8A depicts the skin treated with coumarin-6 dye alone and Fig. 8B depicts transdermal penetration of coumarin-6 dye loaded-DE formulation across the skin barrier. The confocal images demonstrated that the carrier containing the dye was evenly dispersed throughout the stratum corneum, epidermis, and dermis, exhibiting a significantly higher fluorescent intensity. The findings are consistent with prior literature indicating that the utilization of a flexible vesicular system is significant in enhancing drug solubility and optimizing the distribution of the drug molecule into skin tissue^69^.

### Dermal kinetic studies

Dermatokinetic investigations were conducted to assess the dermal pharmacokinetics therapeutic efficacy of the newly formulated DE-gel in comparison to the conventional gel in distinct skin compartments, specifically the epidermis and dermis. Figure 8C and D demonstrate a difference in the permeation of ETO concentration between the epidermal and dermal skin layers following a single application of conventional gel and DE-gel. This observation indicates that the drug concentration over time followed the principles of a one-compartment open body model (1CBM) through the use of dermatokinetic modelling. The administration of DE-gel loaded with ETO exhibited a statistically significant increase (*p* < 0.05) in the transdermal delivery when compared to the conventional gel.

Table 2 displays the values of the following parameters like AUC_0–12 h_ (µg/ cm^2^), ${C}_{max}^{Skin}$(µg/cm^2^), ${T}_{max}^{Skin}$(h) and K_p_ (h^−1^). It is evocative from the results that the duration of stay of the drug was strongly augmented by the developed DE-gel formulation in deep skin layers and ${T}_{max}^{Skin}$(h) decreased markedly. The lower T_max_ by developed formulation vis-à-vis control signifies better penetration and assures faster onset of action. Apart from this ${C}_{max}^{Skin}$ in both the layers i.e., dermal and epidermal and AUC in dermis increased significantly. Therefore, the data ratified that the DE-gel formulation has prospective outcome in regard to enhanced delivery of ETO across the skin in comparison to the conventional formulation.

**Table 2:** Various dermatokinetic parameters (Mean ± SD) of ETO topical formulations in epidermis and dermis.

Parameters	Conventional gel formulation (Mean ± SD)		DE-gel formulation (Mean ± SD)	
	Epidermis	Dermis	Epidermis	Dermis
AUC_0-12 h_ (µg/cm^2^)	623.13 ± 9.3	365.41 ± 18.1	1758.17 ± 42.9	1206.11 ± 89
${C}_{max}^{Skin}$(µg/cm^2^)	96.64 ± 1.61	48.54 ± 2.1	275.66 ± 13.12	138.25 ± 10.11
${T}_{max}^{Skin}$(h)	4.2 ± 0.34	6.1 ± 0.26	3.6 ± 0.39	4.8 ± 0.41
K_p_ (h^−1^)	0.42 ± 0.06	0.32 ± 0.04	0.61 ± 0.09	0.45 ± 0.11

### In vitro cell culture analysis

#### MTT assay

**Figure 9:**
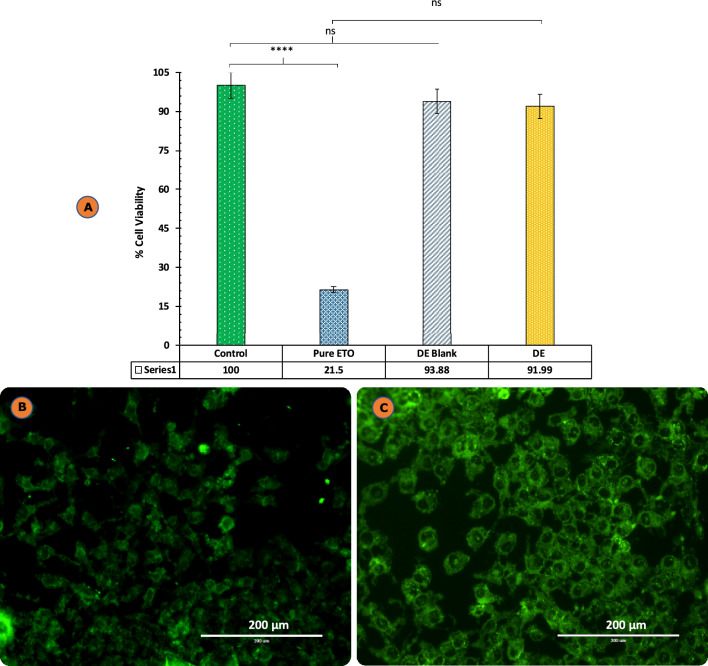
(**A**) Cell viability assay results after 48 h of treatment of pure drug ETO, ETO-DE, blank DE. The results are expressed as % cell viability, taking the viability of control as 100%. Each cross bar indicates ± SD (n = 4;****p < 0.0001; ns-non significant); (**B**) Images depict HaCaT cells subjected to treatment with coumarin-6 alone at 40 ×; and (**C**) Coumarin-6 loaded DE at 40 × objective, respectively, as observed through fluorescent microscopy.

The cell viability assay results using MTT dye are depicted in Fig. 9A. HaCaT cells were used to study the cytotoxic possibility of the formulations. The % viability of untreated cells which served as the control was considered to be 100%. The developed formulation i.e., DE and comparative blank DE without ETO did not show any toxicity till 100 µg/mL after 48 h treatment with almost 91.99% and 93.88% viability. In contrast, the pure drug, namely ETO, demonstrated notable toxicity, resulting in less than 21.5% viability in comparison to the control. The findings confirmed that the formulations developed in-house exhibit no cytotoxic effects on HaCaT cells. Although there was no statistically significant variance in the percentage of cell viability comparing the control group and the created formulations, there was a statistically significant variance between the control group and the pure medication (*p* < 0.05). The observed phenomenon may be attributed to the biocompatible properties inherent in phospholipids.

#### Determination of cellular uptake

The findings of the investigations on cellular uptake portray the internalization of Coumarin-6 loaded DE formulation by HaCaT cells. As demonstrated in Fig. 9C, the cellular uptake assay of Coumarin-6 DE revealed the proficient internalization of the formulation within the cytosol of HaCaT cells within a time frame of 3 h. The test formulations were observed to induce a distinct green fluorescence in the nuclei of cells, as evidenced by the visualization of coumarin-6. Similarly, coumarin-6 dye labelling alone demonstrated the efficient assimilation of the developed formulations by keratinocytes, as illustrated by the green fluorescence in Fig. 9B.

### Stability studies

#### Chemical stability

**Figure 10:**
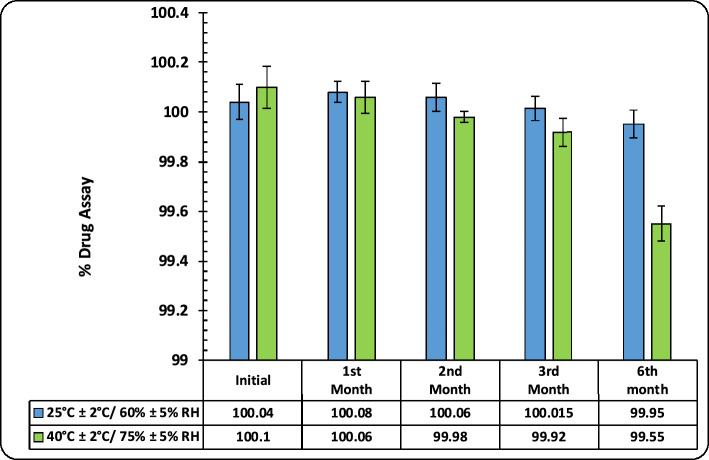
Figure depicting the % drug assay of DE-gel at different storage conditions and time intervals.

Results of stability testing of the developed formulations indicated all the formulations were stable. As discerned from the results given in Fig. 10, the ETO loaded DE-gel formulation is stable at all the studied storage conditions for 6 months. The effect of temperature on the drug migration from one phase to other was not substantial and was suited for topical products.

Effect of temperature on the drug content was found to be insignificant at high temperature conditions (40 °C ± 2 °C/75% ± 5% RH) which can be ascribed to the bilayer packing alteration of vesicles at high temperatures. Similarly, the DE-gel formulation stored at controlled room temperature (25 °C ± 2 °C/60% ± 5% RH) conditions also showed better stability for the studied period.

#### Physical stability

The observations for a period of 6 months were recorded for various physical parameters are enlisted in Table 3**.** The DE formulation showed macroscopic stability on the studied parameters for 6 months at 25 °C ± 2 °C/60% ± 5% RH and 40 °C ± 2 °C/75% ± 5% RH storage conditions. The DE-gel formulation was devoid of notable discoloration and change in odour. The gel consistency also remained good with absence of drug crystals and phase separation. The particle size alteration was below 13%, indicating acceptable variation. However, the present study is limited in scope w.r.t. the changes in the lamellae and viscosity.

**Table 3:** Physical stability assessment studies on DE gel formulation.

Parameter	6 Months	
	Controlled temperature	40 ± 2 °C/75% ± 5% RH
Physical appearance	No discolouration	No discolouration
Odour	No change	No change
Formation of drug crystals	Nil	Nil
Gel consistency	No change	No change
Phase separation	Nil	Nil
pH	6.34 ± 0.16	6.21 ± 0.11

### Efficacy assessment on animal models

#### Skin compliance studies

**Figure 11:**
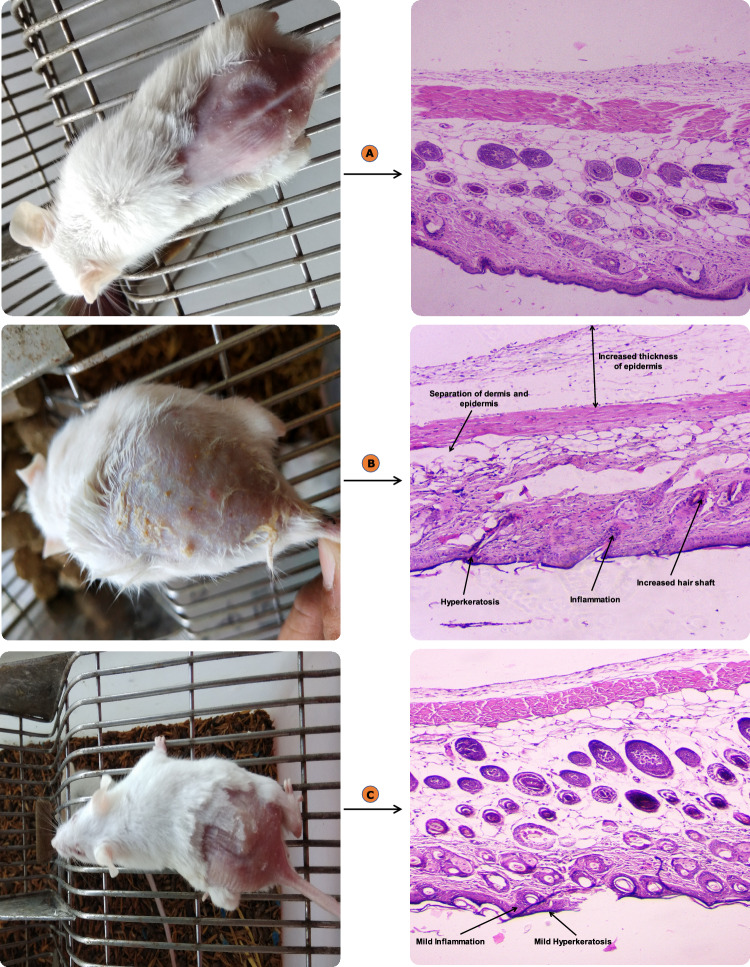
Shows the animals and histology at the end of seven days (**A**) untreated animal (**B**) conventional gel formulation (**C**) DE-gel formulation.

The developed DE-gel formulation was evaluated for any irritating effect on the skin. The erythemal grading (ranging from 0 to 4) were recorded for 7 days. Absence of erythema on skin was observed in case of DE-gel formulation, whereas moderate to severe erythema (light red) scores were observed in case of conventional product as shown in Fig. 11 and the scoring is tabulated in Table 4.

**Table 4:** Mean erythemal scores observed for group, (A) untreated, (B) conventional gel formulation, and (C) DE-gel formulation for 7 days.

Group	Mean erythemal score (Days)						
	1	2	3	4	5	6	7
Untreated	0	0	0	0	0	0	0
Conventional gel	0	0	1	1	2	3	4
DE-gel	0	0	0	0	0	1	1

SCORING: 0 = No erythema, 1 = Slight erythema (light pink), 2 = Moderate erythema (dark pink), 3 = Moderate to severe erythema (light red), 4 = Severe erythema (dark red).

In conformity with the histopathology, the skin section of various animal groups treated with DE-gel formulation and conventional gel formulation were stained with eosin-hematoxylin and evaluated for the histological changes occurred during the period of exposure. Figure 11A showed the photograph of untreated skin, which was normal. Figure 11B showed epidermal thickening and inflammation in the dermis layer on the skin treated with conventional gel. Therefore, the microscopic examination directed that the viable formulation had compromised the normal healthy skin. Furthermore, the skin section treated with DE-gel formulation was found to be healthy with no inflammation in the dermis tissue. It revealed DE-gel did not damage the normal healthy skin Fig. 11C. The study's findings demonstrated the safety and effectiveness of biocompatible phospholipid in the DE-gel system. These positive outcomes can be due to phospholipids interaction with skin components and their ability to establish a skin-depot. The study reported here align with prior literature, which suggests that lipid-based formulations are both safer and more compatible with the skin^55,56^.

#### Anti-inflammatory assessment

##### Xylene-induced ear edema model

**Figure 12:**
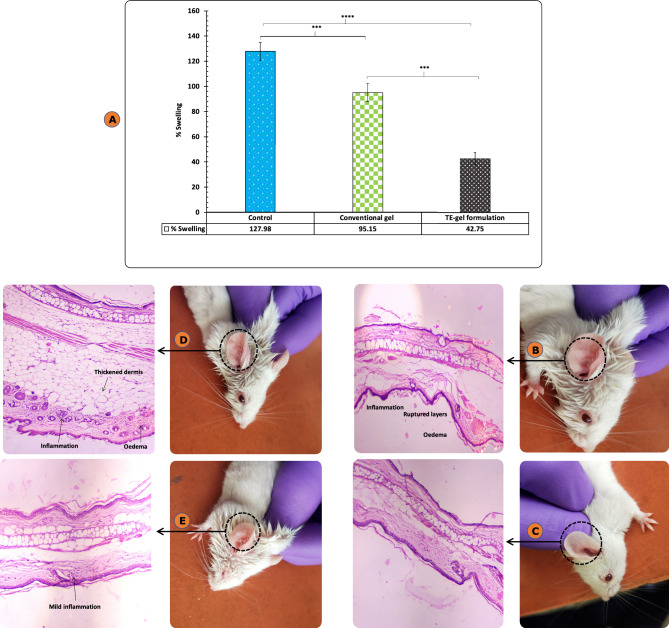
(**A**) Comparison of present ear swelling after application of conventional gel and DE-gel formulations. Each cross bar indicates ± SD (n = 4;***p = 0.001; ****p < 0.0001); (**B**) Histopathology of disease control ear; (**C**) histopathology of normal ear; (**D**) histopathology of conventional gel formulation treated ear; (**E**) histopathology of DE-gel formulation treated ear.

Figure 12A showed DE-gel formulation exhibited remarkably advanced anti-inflammatory activity versus conventional formulation. The % swelling of treated ear was reduced by 2.99 times (DE-gel formulation) and 1.33 times (conventional gel formulation) with respect to untreated ear. Thus, the efficacy of the formulated DE-gel was significantly 2.2-folds higher than that of conventional (*p* < 0.01). The outcomes of the animal study conducted exposed the edge of the vesicular delivery systems as compared to the conventional systems. This accredited to their better interaction with the skin and skin-depot forming potential. As per the histopathological studies, there was division into three groups viz*.* disease control causing swelling, epidermis stretching and detachment of epidermis from dermis as seen in Fig. 12B. The histopathology of untreated ear as shown in Fig. 12C was normal whereas Fig. 12E displayed DE-gel group with healing of ear with intact epidermal and dermal layers and no edema formation. In contrast, Fig. 12D was the group treated with conventional gel formulation reporting incomplete recovery which was manifested from histopathology where disordered articular cartilage with greater number of inflammatory cells was experienced.

##### Anti-arthritic activity: CFA induced arthritis in wistar rats

**Figure 13:**
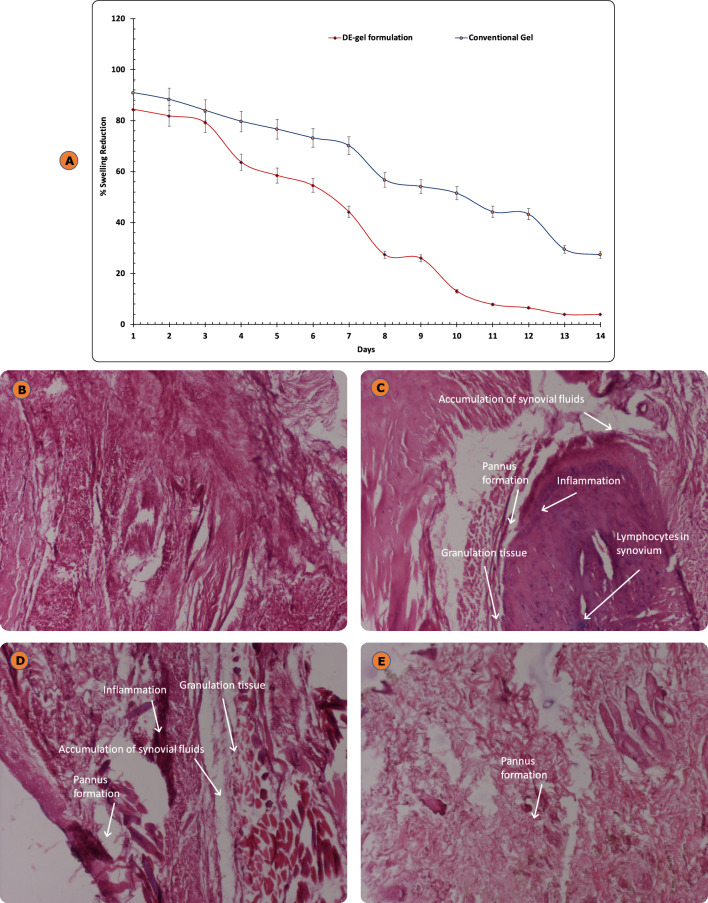
(**A**) Comparison of % arthritis swelling after application of DE-gel formulation and conventional gel formulations. Each cross bar indicates ± SD (n = 4); (**B**) Histopathology of normal paw joint; (**C**) Disease control paw joint; (**D**) conventional gel formulation treated paw joint; (**E**) DE-gel formulation treated paw joint.

To understand the anti-arthritic activity in diseased rats after topical application of DE-gel formulation, the most vital parameter that is % arthritis swelling reduction was calculated. A significant rise in swelling was measured for CFA rats which didn’t receive any treatment. In the conventional gel, a slight reduction in % swelling was detected (i.e., 27.27%), however in the DE-gel formulation, a significant decrease in swelling was noted (i.e., 3.89%), as depicted in Fig. 13A. Thus, efficacy of the DE-gel formulation was sevenfold higher than that of group treated with conventional gel formulation (*p* < 0.01). This shows the superior activity of ETO loaded DE-gel over conventional gel in arthritis and indicated better penetration of drug to the site of action.

The histological examination of paw joint and paw skin of animals suffering from arthritis treated with different ETO formulations was executed to evaluate the level of inflammation and morphological behaviour in the internal structure of the paw joint and paw skin.

Figure 13C represents paw joint of CFA (untreated) induced arthritic rat experienced accretion of synovial fluid, lymphocytes in synovium, tissues granulation along with formation of pannus adding up to the high level of inflammation extended into joint synovium recorded as compared to the normal joint (Fig. 13B). Figure 13D presents a little lower inflammation with the joint space surrounded by inflammatory cells with moderate pannus formation and accretion of synovial fluid in the case of paw joints treated with conventional gel formulation. Almost no signs of inflammation were observed in the paw joint treated with DE-gel formulation attaining improved joint bone health to its normal structure as seen in Fig. 13E.

**Figure 14:**
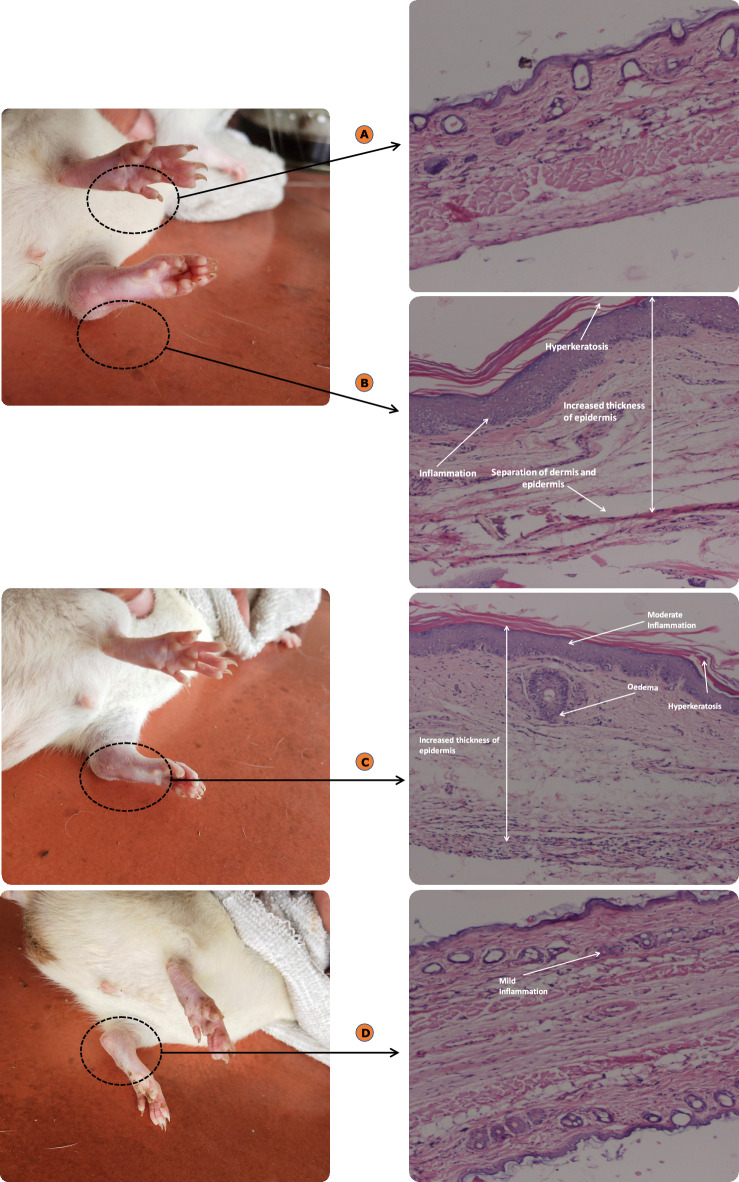
(**A**) Histopathology of normal paw; (**B**) Disease control paw; (**C**) conventional gel formulation treated paw; (**D**) DE-gel formulation treated paw.

Similar results were recorded for the infected paw skin in Fig. 14 further divided into 4 groups i.e., control, untreated, conventional gel, DE-gel treated rat. Figure 14B showed acute inflammation, augmented thickness of the layers, disrupted layers with separation and hyperkeratosis in the untreated rat paw skin. The normal paw skin was observed to be intact having the natural anatomy of skin (Fig. 14A). In comparison to the rat paw skin treated with conventional gel which showed moderate inflammation with slightly higher thickness of the skin layers, edema and hyperkeratosis in Fig. 14C vis-à-vis negligible level of inflammation was detected in case of DE-gel treated rat skin as shown in Fig. 14D.

The severity degree for treated groups is described as a score of inflammation and arthritis from 0 to +  +  +  + (0 indicates normal & +  +  +  + indicates severe). The severity of inflammations for paw skin was observed in the following order: CFA (untreated > conventional gel formulation > DE-gel formulation = control (normal rat).

## Conclusions

The current research endeavor was undertaken to enhance the topical administration attributes of etodolac by investigating the potential of deformable-emulsomes formulated with sodium deoxycholate (SDC). The selection of SDC as the novel excipient was based on its biocompatibility and the flexibility enhancement potential that can translate into enhanced permeability of bioactive substances across the skin. The system that was developed exhibited superior characteristics in terms of ex vivo permeation profile, drug retention, and in vivo anti-inflammatory activity. This can be attributed to the enhanced deformability of the developed vesicles compared to the conventional formulations. As a result, these vesicles were able to effectively penetrate deeper layers of the skin. FESEM, CLSM and dermatokinetic investigations revealed that the flexible vesicles generated were spherical, homogenous and fairly dispersed the drug across the subcutaneous, epidermis, and dermis.

In conclusion, the current research confirmed that the formulation of deformable emulsomes alone and it's gel both can effectively promote the localization of the drug at the site of action in the desired amounts. The methodology that has been implemented has facilitated the development of an effective and regulatory compliant formulation of etodolac for topical usage. The outcomes from this investigation are highly promising and indicate that these biocompatible carriers have the potential to be utilized as a therapeutic approach for managing localized inflammatory disorders such as arthritis. Furthermore, it is anticipated that the findings from this research can be extended to pre-clinical studies in the near future.

## Data Availability

All data generated or analysed during this study are included in this published article.
